# An Online Detection and Rejection Method for Consecutive Outliers in Underwater Long-Baseline Positioning Based on Kinematic Constraints

**DOI:** 10.3390/s26134013

**Published:** 2026-06-24

**Authors:** Le Wang, Jun Su, Runze Mao, Sha Wang

**Affiliations:** Ocean College, Jiangsu University of Science and Technology, Zhenjiang 212003, China; 232211803131@stu.just.edu.cn (L.W.); mao.rz1993@just.edu.cn (R.M.); wangs@just.edu.cn (S.W.)

**Keywords:** underwater long-baseline positioning, outlier detection and rejection, kinematic constraints, interacting multiple model

## Abstract

**Highlights:**

**What are the main findings?**

- Kinematic constraints and multi-step historical observations are utilized for the precise detection of continuous outliers.
- A new matrix is introduced to instantaneously isolate contaminated observations during the measurement update phase of the Kalman filter.

**What are the implications of the main findings?**

- This paper investigates the online detection and rejection method of continuous outliers for long-baseline positioning systems in complex underwater environments.

**Abstract:**

To address the issue of persistent high-magnitude outlier interference affecting long-baseline (LBL) positioning systems in complex marine environments, this paper proposes a kinematic constraint-based Robust Interacting Multiple Model Kalman Filter algorithm. Combined with anchor point initialization and multi-step historical observations, the algorithm constructs a spatial Euclidean distance discriminant criterion. By further incorporating the maximum velocity constraint of the Autonomous Underwater Vehicle (AUV), dynamic decision thresholds are established, and final detection decisions are output to the positioning system. Within the Kalman Filter recursion process, a measurement mask matrix is introduced to instantly isolate measurement outliers, preventing abnormal data from participating in state updates and model probability evolution. Simulation results demonstrate that, compared with standard LBL positioning, conventional single outlier detection, and the conventional maximum correntropy criterion-based Kalman filter (MCC-KF) algorithm, the proposed approach enhances outlier identification and suppression—particularly under consecutive anomaly conditions—thereby improving the positioning accuracy of maneuvering targets in complex underwater scenarios.

## 1. Introduction

Underwater Long-Baseline (LBL) acoustic positioning systems have become a cornerstone for deep-sea resource exploration and collaborative navigation of Autonomous Underwater Vehicles (AUVs) due to their high positioning accuracy, time-invariant error characteristics, and extensive coverage [1,2,3]. The LBL positioning system operates by measuring the one-way travel time (OWTT) of acoustic signals between the target and an array of seafloor-deployed beacons to derive slant range information. Subsequently, the three-dimensional spatial coordinates of the target are reconstructed by employing a spatial spherical intersection model [4,5]. In practical engineering scenarios, fusing LBL positioning measurements within a filtering and tracking framework enables real-time estimation and prediction of target dynamic trajectories. This capability is critical to guarantee the safety of complex underwater operations and improve mission completion efficiency [6,7,8].

However, target positioning and filtering based on the LBL measurement still encounter two major difficulties in complex underwater environments. On the one hand, adverse ocean conditions severely degrade the quality of acoustic measurement data. Restricted by complex deep-sea channel characteristics such as multipath propagation and impulsive noise interference, LBL systems sometimes struggle to acquire accurate time delay measurements, thereby producing a large number of outliers [9,10]. In addition, signal attenuation and packet loss often reduce the number of effective seafloor beacons. In this case, the spatial intersection model fails to calculate a unique target position, resulting in sharp fluctuations of positioning data [11]. On the other hand, underwater targets typically possess maneuvering capability. When executing reconnaissance and obstacle avoidance missions, AUVs frequently switch among multiple motion modes, including uniform straight flight, coordinated turning, and high-amplitude maneuvering [12,13]. For this reason, traditional Kalman filters are difficult to apply directly and effectively in LBL positioning systems for outlier detection and suppression.

In the existing literature, outlier detection and mitigation for underwater positioning systems have been predominantly developed for isolated single outliers, without fully addressing complex underwater conditions involving consecutive dense outliers. The literature by [14] proposed a robust outlier detection and classification method for underwater USBL positioning systems based on causal median filtering. The method constructs adaptive thresholds utilizing the median absolute deviation (MAD), incorporates a four-level classification flag, and applies a time-window reset mechanism to mitigate prolonged data outages, thereby achieving real-time outlier identification independent of navigation filters. It is characterized by a straightforward structure, ease of implementation, and robust performance against random anomalies and brief data losses. Nevertheless, its efficacy remains constrained by the static nature of the window lengths and thresholds, which can limit its adaptability and reduce detection accuracy when encountering dense, consecutive outliers. To address the issues of measurement outliers and missing values in the ultra-short baseline (USBL) underwater positioning system, the literature by [15] presents a robust data cleaning method based on online support vector regression. It constructs time-series samples via a sliding window to implement one-step-ahead prediction, thereby realizing outlier replacement and missing value imputation. This purely data-driven approach delivers outstanding real-time performance and can handle continuous outliers; however, it is only applicable to rectilinear target motion scenarios.

Beyond these heuristic or data-driven methods, modern robust Bayesian estimation frameworks have gained significant attention for their ability to handle non-Gaussian and heavy-tailed noise. In particular, the Maximum Correntropy Criterion (MCC) has emerged as an effective tool for suppressing impulsive noise by utilizing a local similarity measure. Jwo et al. [16] developed a robust navigation filter based on MCC with an adaptive kernel bandwidth (AKB), which dynamically adjusts the kernel width to enhance the filter’s resilience against anomalous values while maintaining high accuracy in Gaussian environments. Furthermore, in the field of autonomous underwater vehicle (AUV) navigation, Stepanov et al. [17] investigated a correntropy-based extended Kalman filter and a recursive rejection procedure. Their approach identifies outliers by comparing filter residuals with the diagonal elements of the measurement prediction error covariance matrix, providing a robust solution for collaborative navigation when distance measurements are contaminated. However, the practical performance of these correntropy-based algorithms remains affected by the choice of kernel bandwidth.

The Interacting Multiple Model (IMM) has emerged as a mature framework for underwater maneuvering target localization and filtering [18,19]. Featuring excellent stability and strong robustness against model mismatch, this algorithm achieves an optimal trade-off between computational complexity and tracking accuracy, thereby being widely recognized as the preferred solution for such applications [20]. Combined with the Kalman filter, IMM enables the optimization of available target localization data while supporting the integration of outlier detection and rejection modules.

To overcome the limitation that existing algorithms cannot strike a balance between continuous outlier processing and target maneuver adaptation, a Kinematic-Constrained Robust IMM-KF (KC-RIMM-KF) algorithm incorporating kinematic constraints and indicator functions is developed in this paper. Departing from the conventional single-residual statistical testing strategy, the algorithm integrates target kinematic constraints with historical positioning data for anomaly detection. By incorporating a Measurement Mask Matrix, the KC-RIMM-KF achieves instantaneous isolation of degraded observations within the IMM-KF recursive loop, ensuring that contaminated data do not participate in state updates or model probability evolution. In this work, the IMM-KF algorithm is adopted to leverage the target’s maneuvering model for accurate outlier detection. The core innovation of this paper lies in the precise identification of continuous outliers. In particular, the proposed method can effectively prevent normal measurements from being misjudged as outliers and achieves better real-time performance. Monte Carlo simulations are performed across two typical underwater scenarios characterized by standard-intensity and high-intensity maneuvering. Results demonstrate that the proposed algorithm preserves stable tracking under consecutive outlier disturbances. While both the MCC-KF and the proposed method yield smaller positioning errors than the traditional single-point detection method and the raw LBL positioning scheme, the proposed algorithm achieves a faster convergence back to the baseline level once the outliers disappear.

Notably, the core distinction between the proposed KC-RIMM-KF and existing robust IMM-KF frameworks lies in the outlier detection paradigm. Most conventional robust IMM variants rely on single-step filter residuals or correntropy-based weight scaling for anomaly detection, and their performance degrades sharply under consecutive dense outliers as residuals become gradually contaminated. In contrast, our method constructs an independent detection pipeline based on kinematic constraints and multi-step historical anchor observations, identifying anomalies via spatial Euclidean distance decoupled from the filter state. This design eliminates reliance on residual statistics, ensures reliable detection of persistent outliers, and enables faster accuracy recovery after anomalies cease while preserving stable multi-model fusion during target maneuvers.

## 2. Methods for Long-Baseline Positioning Solution

The Long-Baseline positioning system estimates the target’s position based on a spatial distance intersection model by deploying multiple acoustic beacons on the seabed and measuring the one-way travel time of acoustic signals from the target to each beacon. Assuming the system employs a synchronous positioning architecture with a fixed update interval T, this section derives the linear and nonlinear spatial positioning models of the LBL system and analyzes the generation mechanism of positioning outliers from observation dimension.

### 2.1. Basic Spatial Spherical Intersection Positioning Model

**Figure 1:**
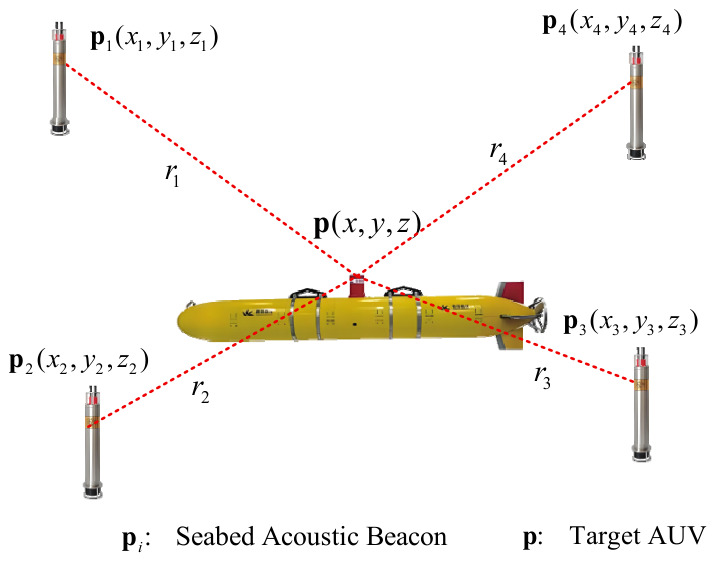
Schematic diagram of the spatial spherical intersection geometry for LBL positioning.

The fundamental principle of LBL positioning relies on spatial geometric intersection [21]. The absolute slant range from the target to each beacon can be deduced from the propagation delay of the acoustic signal, where each range measurement corresponds to a sphere in 3D space centered at the beacon’s coordinates. The principle of distance intersection is illustrated in Figure 1.

Let the coordinate vector of the i-th beacon in the system be $p_{i}={\left[x_{i},y_{i},z_{i}\right]}^{T}$, and the target coordinate vector to be estimated be $p={\left[x,y,z\right]}^{T}$. Assuming the equivalent underwater sound speed is c, and the measured OWTT is $\tau_{i}$, the corresponding measured slant range is $r_{i}=c\tau_{i}$. By introducing the L2-norm representation of vectors, the basic spherical observation equation is established as:

(1) $$\|p-p_{i}{\|}^{2}=r_{i}^{2},\;i=1,2,\ldots ,M\;$$

According to the principles of spatial analytic geometry, the intersection of three non-collinear spheres generally yields two intersecting points, whereas the intersection of four spheres is required to uniquely determine the spatial coordinates. Considering a system comprising M (M≥4) beacons, the nonlinear quadratic terms can be eliminated by subtracting the observation equation of the 1st beacon from those of the 2nd, 3rd, ⋯, M-th beacons, respectively. Consequently, the original model can be transformed into the following linear algebraic matrix form:

(2) Ap=b 

where the target position vector is $p={\left[x,y,z\right]}^{T}$. The state coefficient matrix A and the observation vector b are constructed as follows:

(3) $$A=2\left[\begin{array}{c} {\left(p_{2}-p_{1}\right)}^{T}\\ {\left(p_{3}-p_{1}\right)}^{T}\\ \vdots \\ {\left(p_{M}-p_{1}\right)}^{T} \end{array}\right]=2\left[\begin{array}{ccc} x_{2}-x_{1} & y_{2}-y_{1} & z_{2}-z_{1}\\ x_{3}-x_{1} & y_{3}-y_{1} & z_{3}-z_{1}\\ \vdots & \vdots & \vdots \\ x_{M}-x_{1} & y_{M}-y_{1} & z_{M}-z_{1} \end{array}\right]\;$$

(4) $$b=\left[\begin{array}{c} \|p_{2}{\|}^{2}-\|p_{1}{\|}^{2}+r_{1}^{2}-r_{2}^{2}\\ \|p_{3}{\|}^{2}-\|p_{1}{\|}^{2}+r_{1}^{2}-r_{3}^{2}\\ \vdots \\ \|p_{M}{\|}^{2}-\|p_{1}{\|}^{2}+r_{1}^{2}-r_{M}^{2} \end{array}\right]\;$$

where $\|p_{i}{\|}^{2}=x_{i}^{2}+y_{i}^{2}+z_{i}^{2}$ denotes the squared distance from the i-th beacon to the origin of the coordinate system. When the state matrix A satisfies the full column rank condition, the 3D coordinate estimate of the target can be directly obtained via least squares estimation, i.e., $p={\left(A^{T}A\right)}^{-1}A^{T}b$.

### 2.2. Depth-Constrained 2D Positioning Model

In practical engineering applications, if the underwater vehicle is equipped with a high-precision depth sensor (where the known target depth z=h serves as a prior constraint), the original 3D spatial spherical intersection model is reduced to a 2D circular intersection model. Consequently, the coefficient matrix A is correspondingly reduced in dimension, and the state vector to be solved is simplified to $p_{2D}={\left[x,y\right]}^{T}$. The linearized equations can be rewritten as:

(5) $$2\left[\begin{array}{cc} x_{2}-x_{1} & y_{2}-y_{1}\\ x_{3}-x_{1} & y_{3}-y_{1}\\ \vdots & \vdots \\ x_{M}-x_{1} & y_{M}-y_{1} \end{array}\right]p_{2D}=\left[\begin{array}{c} \left\|p_{2}{\|}^{2}-\|p_{1}{\|}^{2}+r_{1}^{2}-r_{2}^{2}-2h(z_{2}-z_{1}\right)\\ \left\|p_{3}{\|}^{2}-\|p_{1}{\|}^{2}+r_{1}^{2}-r_{3}^{2}-2h(z_{3}-z_{1}\right)\\ \vdots \\ \left\|p_{M}{\|}^{2}-\|p_{1}{\|}^{2}+r_{1}^{2}-r_{M}^{2}-2h(z_{M}-z_{1}\right) \end{array}\right]\;$$

Denoting the aforementioned dimension-reduced overdetermined equation system as $A_{2D}p_{2D}=b_{2D}$, the optimal estimate of the target’s horizontal position can be solved using the Least Squares method when the number of beacons providing valid observation data is M≥3:

(6) $$p_{2D}={\left(A_{2D}^{T}A_{2D}\right)}^{-1}A_{2D}^{T}b_{2D}\;$$

Conversely, if a nonlinear analytical method is directly employed without linearization, the combination of two spheres and the known depth plane will produce an ambiguous dual solution regarding (x,y) in 3D space. To eliminate this pseudo-solution, the range observation equation from a third beacon must be introduced, or it must be combined with the dynamic trajectory constraints from the previous epoch to determine the true coordinates uniquely.

### 2.3. Analysis of Outlier Generation

Synthesizing the mathematical models above, it is evident that the positioning reliability of the LBL system highly depends on the number of valid observation beacons (i.e., the observation redundancy of the system). A standard four-beacon LBL system achieves a unique estimate of the target position by constructing a redundant observation equation system, and its positioning robustness is closely correlated with the number of observation beacons.

When a single beacon in the system experiences a time delay measurement anomaly or coordinate calibration deviation, this anomalous beacon can be directly excluded. After excluding a single beacon, the remaining three beacons can still construct three independent distance equations. Although the system degenerates into a nonlinear spherical equation system and yields a dual solution at this stage, the unique solution can be determined simply by combining depth information or trajectory constraints. The system continues to position normally and will not induce outliers.

However, when two beacons in the system exhibit anomalies simultaneously, the number of valid observation beacons drops to two, allowing for the construction of only two independent distance observation equations. According to the principle of spherical intersection, the intersection of two spheres forms a circle in 3D space, which renders the equation system severely under-determined and makes it impossible to determine the 3D coordinates of the target uniquely. Under such extreme geometric observation degradation, if the system forcibly executes the calculation using conventional algorithms, it will output a positioning result that significantly deviates from the true physical position. This abrupt deviation constitutes the positioning outlier. Beyond the observation degradation discussed above, underwater positioning outliers are also driven by acoustic physical factors such as multipath effects from surface/bottom reflections, sound speed profile fluctuations caused by temperature and salinity variations, and signal detection errors in high-noise environments. Outliers in underwater positioning systems are fundamentally triggered by underwater acoustic channel disturbances and insufficient observation redundancy.

It is worth noting that the positioning and tracking framework proposed in this paper adopts a two-stage processing architecture—where the spatial coordinates are first resolved geometrically and then input into the subsequent filtering and outlier rejection stage—rather than directly feeding raw range measurements into an Extended Kalman Filter (EKF).

In practical underwater environments, clock synchronization mismatches and spatiotemporal variations in sound speed introduce systematic biases into both travel time measurements and sound speed estimates. These biases not only significantly degrade positioning accuracy but also further induce measurement outliers. To mitigate these issues, preprocessing workflows are widely implemented in engineering practice. First, high-precision atomic clocks are calibrated against GPS time prior to deployment to minimize clock synchronization errors between the underwater vehicle and the beacons. Second, to address sound ray bending caused by non-uniform sound velocity profiles (SVP), a ray-tracing technique is applied to compensate for propagation delays and correct slant ranges based on measured SVP data. This paper focuses on detecting and eliminating existing outliers using the IMM-KF filter combined with historical measurement data, and does not elaborate on the specific underlying causes of outlier occurrence.

The primary justification for this design lies in the reference benchmark required for outlier detection. The outlier discrimination scheme relies on target position information as part of its judgment criterion. In the absence of outliers, the LBL positioning system features high positioning accuracy, and its algebraic coordinate solutions can serve as a reliable, model-independent reference. Conversely, if raw range measurements are directly input into an EKF for joint estimation and rejection, the outlier detection logic becomes heavily dependent on the filter’s state predictions. In maneuvering target tracking scenarios, transient motion model mismatches can cause prediction errors that are substantially larger than the nominal measurement noise of the LBL system. Relying solely on EKF predictions under such conditions may lead to false rejection of valid measurements or filter divergence. By resolving the coordinates first, we obtain a baseline that is decoupled from the dynamic model, thereby improving the robustness of outlier discrimination during target maneuvers.

## 3. Robust IMM-KF Algorithm Based on Kinematic Constraints and Indicator Functions

Aiming at the limited applicability of existing outlier elimination methods for underwater LBL positioning systems, this paper proposes an online detection and processing method for continuous outliers based on Euclidean distance kinematic constraints. Taking stable positioning data collected in the initial phase as anchor points, the method establishes the initial target trajectory. By integrating Euclidean distance constraints of target motion characteristics with historical positioning data, it realizes anomaly discrimination, thereby efficiently and accurately identifying the occurrence time and duration of continuous outliers.

On this basis, the proposed continuous outlier detection and processing method is embedded into the IMM-KF framework, and a robust Interacting Multiple Model Kalman Filter algorithm is constructed. This algorithm enables simultaneous positioning and filtering coordinated with the Kalman filter and the positioning system. When positioning observations at a single time step or multiple consecutive time steps are detected and classified as outliers, the algorithm can automatically switch to Kalman filter predictions to replace abnormal values and complete positioning correction.

### 3.1. Target Kinematic Modeling and Observation Description

Assume that the motion process of the underwater target can be covered by l typical models [22]. The discrete-time state-space equation for the j-th model (j=1,…,l) is defined as:

(7) $$X_{j}(k)=\Phi_{j}(k-1)X_{j}(k-1)+G_{j}(k-1)W_{j}(k-1)\;$$

where the state vector $X_{j}(k)$ comprises the 3D position and velocity of the target; $\Phi_{j}(k-1)$ represents the state transition matrix of the j-th model; $G_{j}(k-1)$ represents the process noise coupling matrix of the j-th model; and $W_{j}(k-1)\sim N(0,Q_{j})$ represents the process noise vector with covariance matrix $Q_{j}$. The corresponding 3D position measurement equation is defined as:

(8) Z(k)=HX(k)+v(k)

where Z(k) is the measurement vector, H is the observation matrix, and v(k)~N(0,R) is the measurement noise vector with covariance matrix R.

### 3.2. Outlier Detection Based on Kinematic Constraints

To enable the algorithm to detect consecutive outliers, a multi-step criterion based on Kinematic Constraints is established as follows:

Step 1: Anchor Point Initialization

In the initial stage of the positioning system, the first batch of valid positioning points is selected as initial anchor points, which serve as the reference basis for subsequent outlier detection. The maximum length of consecutive outliers detectable by this method is N−1. In practical engineering, the initial N sets of measurements are collected and verified during the stationary alignment phase before the UUV is launched, and this phase typically lasts for several minutes. Since the vehicle remains stationary throughout this period, anomalous measurements can be identified and eliminated based on the GPS measurements acquired by the UUV prior to submergence. However, the probability of obtaining consecutive normal measurements decreases rapidly as Nincreases. Accordingly, the value of N should not be set too large (typically a small integer, e.g., 5 or 6), so as to avoid excessively prolonging the system initialization phase due to overly stringent detection criteria. Once these N sets of stable measurements are verified and established as the initial anchor points, the multi-step backtracking criterion will be formally activated. The specific data filtering procedure is illustrated in the pseudocode Algorithm 1 below. **Algorithm 1** Anchor Point InitializationInput: Z(k), $Z_{gps}(k)$, γ, $t_{initial}$
Output: $I_{out}$; 1. $N_{q}=0$; 2. $I_{out}=0$; 3. $N_{initial}=floor(t_{initial}/T)$; 4. for i from 1 to $N_{initial}$ do: 5.   $D(k)=\|Z(k)-Z_{gps}(k){\|}_{2}$; 6.     if D(k)≥γ then: 7.     $N_{counter}=0$; 8.     else: 9.     $N_{counter}=N_{counter}+1$; 10.    end 11.     if $N_{counter}==N$; 12.     $I_{out}=1$; 13.     break; 14.     end 15. end 16. return $I_{out}$;

Here, Z(k) denotes the LBL measurement vector at the k-th time instant; $Z_{gps}(k)$ represents the reference GPS measurement vector; $t_{\mathrm{initial}}$ denotes the total pre-allocated initialization and alignment duration; $I_{\mathrm{out}}$ is the initialization status flag where $I_{\mathrm{out}}=1$ indicates the successful establishment of N verified anchor points. The predefined anomaly detection threshold γ is defined as the product of the maximum velocity $v_{\max}$ and the sampling period T, i.e., $\gamma =v_{\max}\cdot T$; and $N_{counter}$ is an auxiliary counter representing the current number of consecutive outlier-free measurements.

Step 2: Spatial Distance Statistic Calculation

Define the spatial Euclidean distance statistic between the current observation Z(k) and the n-th historical valid anchor point Z(k−n) as:

(9) $$D_{n}(k)=\|Z(k)-Z(k-n){\|}_{2},\;n=1,2,\ldots ,N$$

where N denotes the preset maximum number of backtrack steps.

Step 3: Dynamic Rejection Threshold and Relaxation Coefficients

Based on the underwater target maximum velocity limit, a deterministic rejection threshold $\gamma_{n}$ is constructed:

(10) $$\gamma_{n}=v_{\max}\cdot nT$$

where T is the positioning update period.

When the backtrack step n is large, the actual trajectory of the target probably involves maneuvers, making its spatial straight-line distance inevitably smaller than the theoretical threshold $v_{\max}\cdot nT$.

Step 4: Indicator Function Output

Based on the comparison results between the Euclidean distance and the dynamic rejection threshold, an indicator function $I_{\mathrm{index}}(k)$ is defined as the logical discrimination factor [23]:

(11) $$I_{\mathrm{index}}(k)=\left\{\begin{array}{cc} 0, & \forall n\in \{1,\ldots ,N\},D_{n}(k)\geq \gamma_{n}\;(\mathrm{Outlier})\\ 1, & \mathrm{Otherwise}\;(\mathrm{Normal}) \end{array}\right.$$

### 3.3. Robust Recursive Procedure of the KC-RIMM-KF

**Figure 2:**
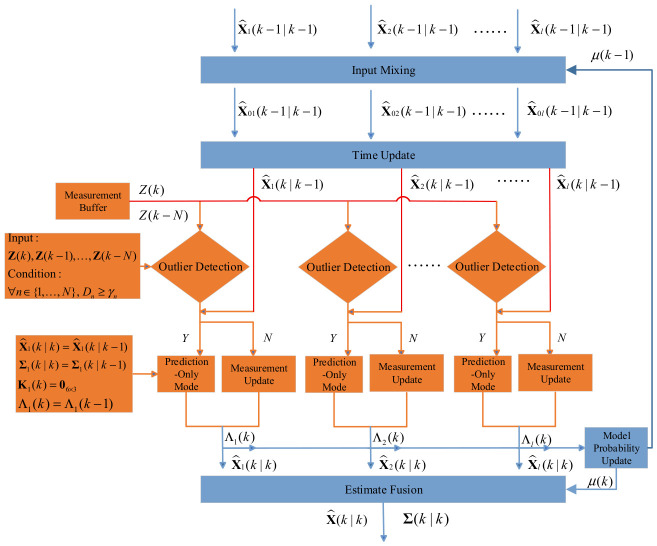
Block diagram of the proposed robust IMM-KF algorithm.

The discrimination factor $I_{\mathrm{index}}(k)$ is embedded into the IMM-KF framework. The single-cycle recursive process (as illustrated in Figure 2) comprises the following stages:

(1) Input Mixing and Time Update

Calculate the mixing probability ${\bar{\varepsilon }}_{j}(k-1)=\sum_{i=1}^{l}p_{ij}\mu_{i}(k-1)$, and deduce the mixed initial state ${\hat{X}}_{0j}(k-1|k-1)$ and mixed covariance $\Sigma_{0j}(k-1|k-1)$ for each sub-filter:

(12) $${\hat{X}}_{0j}(k-1|k-1)=\sum_{i=1}^{l}{\hat{X}}_{i}(k-1|k-1)\frac{p_{ij}\mu_{i}(k-1)}{{\bar{\varepsilon }}_{j}(k-1)}$$

(13) $$\Sigma_{0j}(k-1|k-1)=\sum_{i=1}^{l}\frac{p_{ij}\mu_{i}(k-1)}{{\bar{\varepsilon }}_{j}(k-1)}\left\{\Sigma_{i}(k-1|k-1)+d_{ij}(k-1)d_{ij}^{T}(k-1)\right\}\;$$

where $d_{ij}(k-1)={\hat{X}}_{i}(k-1|k-1)-{\hat{X}}_{0j}(k-1|k-1)$ represents the state difference vector between the i-th sub-filter and the j-th mixed initial state.

(2) Adaptive Measurement Update and Outlier Masking Mechanism

Obtain the predicted state ${\hat{X}}_{j}(k|k-1)$. To eliminate the positioning measurement points identified as outliers, a Measurement Mask Matrix $M(k)=I_{\mathrm{index}}(k)I_{3}$ is introduced. The measurement update is performed as:

(14) $${\hat{X}}_{j}(k|k)={\hat{X}}_{j}(k|k-1)+K_{j}(k)M(k)[Z(k)-H{\hat{X}}_{j}(k|k-1)]$$

(15) $$\Sigma_{j}(k|k)=[I_{n_{x}}-K_{j}(k)M(k)H]\Sigma_{j}(k|k-1)$$

where $K_{j}(k)$ is the sub-filter Kalman gain matrix, which is computed as: $K_{j}(k)=\Sigma_{j}(k|k-1)H^{T}{\left[H\Sigma_{j}(k|k-1)H^{T}+R(k)\right]}^{-1}\;$ with R(k) denoting the measurement noise covariance matrix, and $n_{x}$ representing the state dimension. When $I_{\mathrm{index}}(k)=0$, the innovation feedback is instantaneously nullified, and the filter automatically degrades to a prediction-only mode.

(3) Likelihood Calculation and Model Probability Update

For normal observations, calculate the Gaussian likelihood $\Lambda_{j}(k)$ with measurement dimension m=3:

(16) $$\Lambda_{j}(k)=\frac{1}{{\left(2\pi \right)}^{m/2}\det{\left(S_{j}(k)\right)}^{1/2}}\exp\left\{-\frac{1}{2}\nu_{j}^{T}(k)S_{j}^{-1}(k)\nu_{j}(k)\right\}$$

(17) $$S_{j}(k)=H\Sigma_{j}(k|k-1)H^{T}+R(k)$$

In Equation (16), $\nu_{j}(k)=Z(k)-{\hat{Z}}_{j}(k)$ represents the innovation vector (or residual). If the current observation is identified as an outlier in Step 4, the likelihood collapse is avoided by forcing $\Lambda_{j}(k)=\Lambda_{j}(k-1)$. Subsequently, update the model probability $\mu_{j}(k)$:

(18) $$\mu_{j}(k)=\frac{\Lambda_{j}(k)\sum_{i=1}^{l}p_{ij}\mu_{i}(k-1)}{\sum_{j=1}^{l}\left[\Lambda_{j}(k)\sum_{i=1}^{l}p_{ij}\mu_{i}(k-1)\right]}$$

### 3.4. State Fusion and Adaptive Output Strategy

(1) Global Weighted Fusion

Based on model probabilities $\mu_{j}(k)$, the fused global state estimate $\hat{X}(k|k)$ and the global error covariance Σ(k|k) are calculated:

(19) $$\hat{X}(k|k)=\sum_{j=1}^{l}{\hat{X}}_{j}(k|k)\mu_{j}(k)$$

(20) $$\Sigma (k|k)=\sum_{j=1}^{l}\mu_{j}(k)\left\{\Sigma_{j}(k|k)+[{\hat{X}}_{j}(k|k)-\hat{X}(k|k)]{\left[{\hat{X}}_{j}(k|k)-\hat{X}(k|k)\right]}^{T}\right\}$$

(2) Final Output Switching Strategy

To maintain high precision under normal conditions while ensuring trajectory continuity during interference, the final output $\Psi_{x}(k)$ is determined by the following switching logic:

(21) $$\Psi_{x}(k)=\left\{\begin{array}{ll} H\hat{X}(k|k), & I_{\mathrm{index}}(k)=0\;(\mathrm{Outlier}\;\mathrm{detected},\;\mathrm{use}\;\mathrm{filter}\;\mathrm{estimate})\\ Z(k), & I_{\mathrm{index}}(k)=1\;(\mathrm{Normal}\;\mathrm{observation},\;\mathrm{retain}\;\mathrm{raw}\;\mathrm{LBL}\;\mathrm{data}) \end{array}\right.$$

## 4. Simulation

In this section, numerical simulations are conducted for two typical underwater scenarios, where the target maneuvering intensity and the number of outliers are different. The objective is to systematically validate the robustness of the proposed outlier detection and processing algorithm in the presence of severe consecutive outliers.

### 4.1. System Model Parameters and Performance Metrics

#### 4.1.1. System Matrices and Filter Configuration

The kinematic state of the target is given by

$$X={\left[x\;y\;z\;v_{x}v_{y}v_{z}\right]}^{T}$$

where (x,y,z) represent the target position and $\left(v_{x},v_{y},v_{z}\right)$ is target velocity.

Considering the distinct motion characteristics of underwater vehicles, the IMM method employed in the simulation adopts one Constant Velocity (CV) model and two Coordinated Turn (CT) models [24], where the $F_{\mathrm{CT}}$ model assumes that the horizontal maneuvering is decoupled from the vertical dimension. In typical AUV exploration missions, coordinated turns are primarily executed in the horizontal plane, while the vertical motion is governed by stable depth-keeping or slow ascent/descent maneuvers. Consequently, the vertical components in $F_{\mathrm{CT}}$ are simplified as a linear CV process to reflect this constrained vertical maneuvering pattern.

The state transition matrices for the CV and CT models used in this section are defined as:

$$F_{\mathrm{CV}}=\left[\begin{array}{cc} I_{3} & TI_{3}\\ 0_{3} & I_{3} \end{array}\right],\;F_{\mathrm{CT}}=\left[\begin{array}{cccccc} 1 & 0 & 0 & \frac{\sin\omega T}{\omega } & -\frac{1-\cos\omega T}{\omega } & 0\\ 0 & 1 & 0 & \frac{1-\cos\omega T}{\omega } & \frac{\sin\omega T}{\omega } & 0\\ 0 & 0 & 1 & 0 & 0 & T\\ 0 & 0 & 0 & \cos\omega T & -\sin\omega T & 0\\ 0 & 0 & 0 & \sin\omega T & \cos\omega T & 0\\ 0 & 0 & 0 & 0 & 0 & 1 \end{array}\right]$$

$$G=\left[\begin{array}{c} \frac{T^{2}}{2}I_{3}\\ TI_{3} \end{array}\right]$$

where $I_{3}$ denotes the 3-dimensional identity matrix, $0_{3}$ denotes the 3-dimensional zero matrix, and ω represents the target’s turn rate (angular velocity).

The process noise covariance matrix is set as $Q=\mathrm{diag}([{\left(10^{-2}\right)}^{2},{\left(10^{-2}\right)}^{2},{\left(10^{-3}\right)}^{2}])$. Notably, the noise intensity allocated to the z-axis is an order of magnitude smaller than that of the horizontal axes. This configuration is based on the fact that underwater vehicles are typically equipped with high-precision depth control systems (e.g., pressure-based depth sensors and buoyancy trim systems). These systems ensure that the vertical motion is significantly more predictable and less susceptible to random oceanic disturbances compared to horizontal maneuvering, which is often affected by complex currents and thruster uncertainties.

The observation equation for the LBL positioning system is Z(k)=HX(k)+v(k), with the measurement matrix $H=[I_{3},0_{3}]$. The measurement noise v(k) follows a zero-mean Gaussian distribution with covariance $R=\sigma^{2}I_{3}$. The sampling interval is set to T=3 s, and κ=100 Monte Carlo runs are performed, with the occurrence timing of outliers kept consistent across all runs.

Positioning outliers are generated using a Gaussian distribution with a standard deviation of $\sigma_{w}=50\;m$. The transition probability matrix for the IMM filter is:

$$\Pi =\left[\begin{array}{ccc} 0.95 & 0.025 & 0.025\\ 0.025 & 0.95 & 0.025\\ 0.025 & 0.025 & 0.95 \end{array}\right]$$

The initial model probability is set as $u={\left[0.99,0.005,0.005\right]}^{T}$. The dominance of the first model (0.99) corresponds to the stationary state of the underwater target. In practical underwater operations, the vehicle typically remains in a stationary state at the starting point to facilitate acoustic modem synchronization and inertial sensor alignment before commencing its maneuvering profile.

#### 4.1.2. Outlier Detection Parameters

The number of backtrack steps is set to N=5 in this paper, enabling the algorithm to detect up to N−1=4 consecutive outliers. The proposed algorithm utilizes multi-step spatial distance statistics, $D_{1}$ to $D_{5}$, based on kinematic constraints for online outlier identification. The detection threshold incorporates a decaying attenuation coefficient to constitute a dynamic gate combined with the maximum target velocity $v_{\max}=10\;\mathrm{kn}$. This velocity limit is selected to reflect the typical mobility of high-end Unmanned Underwater Vehicles (UUVs), ensuring that any coordinate jump exceeding this threshold limit can be identified as an outlier.

#### 4.1.3. MCC-KF Parameter Configuration and Formulations

To verify the accuracy of the proposed outlier detection logic under consecutive outliers, the conventional MCC-KF is adopted as a benchmark. This algorithm suppresses outliers by automatically scaling the measurement noise covariance based on the correntropy kernel weight L(k). In this benchmark, the kernel bandwidth $\sigma_{mcc}$ is maintained as a fixed constant.

The core mathematical formulations of the MCC-KF are defined as follows. First, the squared Mahalanobis distance of the measurement innovation vector is computed as:

$$d^{2}(k)={\left[Z(k)-H\hat{X}(k|k-1)\right]}^{T}R^{-1}[Z(k)-H\hat{X}(k|k-1)]$$

where Z(k) represents the observation vector, $\hat{X}(k|k-1)$ is the predicted state, and R denotes the nominal measurement noise covariance matrix.

Subsequently, the correntropy-based kernel weight factor L(k) is derived using a Gaussian kernel function:

$$L(k)=\exp\left(-\frac{d^{2}(k)}{2\sigma_{mcc}^{2}}\right)$$

where the kernel bandwidth $\sigma_{mcc}$ is a constant.

Consequently, the equivalent measurement noise covariance matrix $\tilde{R}(k)$ is reconstructed to restrict the measurement update weight during anomalies:

$$\tilde{R}(k)=\frac{R}{\max(L(k),10^{-4})}$$

The kernel bandwidth is set to a constant value of $\sigma_{mcc}=12$. This parameter is selected to balance the tracking sensitivity under typical measurement noise and the attenuation capability against outlier-contaminated coordinate anomalies.

#### 4.1.4. Performance Metrics

To quantitatively evaluate tracking accuracy and stability, the Root Mean Square Error (RMSE) and Average RMSE (ARMSE) are employed [25]. For 3D positioning, the indices at time step k are calculated as:

$$\mathrm{RMSE}(k)=\sqrt{\frac{1}{\kappa }\sum_{n=1}^{\kappa }\left[{\left(x_{n}(k)-{\hat{x}}_{n}(k)\right)}^{2}+{\left(y_{n}(k)-{\hat{y}}_{n}(k)\right)}^{2}+{\left(z_{n}(k)-{\hat{z}}_{n}(k)\right)}^{2}\right]}$$

$$\mathrm{ARMSE}=\frac{1}{K}\sum_{k=1}^{K}\mathrm{RMSE}(k)$$

where κ denotes the number of Monte Carlo runs, K is the total number of samples, and $\left(x_{n}(k),y_{n}(k),z_{n}(k)\right)$ and $\left({\hat{x}}_{n}(k),{\hat{y}}_{n}(k),{\hat{z}}_{n}(k)\right)$ represent the true and estimated positions at time step k in the n-th run, respectively.

#### 4.1.5. Computational Efficiency and Execution Time

In addition to the positioning accuracy metrics, the computational execution time is another essential factor to ensure online applicability. The proposed algorithm is executed over multiple independent runs to evaluate its computational overhead. Table 1 presents the total filtering execution times of the proposed algorithm under Case 1 and Case 2, alongside those of the benchmark MCC-KF under Case 1 for comparison, relative to their respective entire mission durations.

According to Table 1, the proposed filter requires approximately 0.023 s and 0.035 s to complete the execution under Case 1 and Case 2, respectively, while the benchmark MCC-KF requires approximately 0.0058 s under Case 1. Although the MCC-KF exhibits a lower computational overhead due to its single-filter structure, the execution time of the proposed multi-model algorithm remains on the millisecond scale. Given the total mission times of 300 s and 450 s, the computational cost is minor, which supports its practical implementation in real-time navigation.

### 4.2. Simulation and Result Analysis for Case 1

#### 4.2.1. Case 1 Scenario Description and Outlier Configuration

Case 1 represents a common maneuvering scenario where the target executes a series of maneuvers in 3D space. The initial position is $p_{0}={\left[400\;m,200\;m,100\;m\right]}^{T}$ with a total duration of 300 s. The trajectory sequence consists of six phases: (1) Stationary (0–15 s); (2) Acceleration (16–30 s) with $a={\left[0.15,0.05,0\right]}^{T}{\;m/s}^{2}$; (3) Steady-state cruise (31–60 s); (4) Primary turn (61–165 s, $\omega_{1}\approx 0.015\;\mathrm{rad}/s$); (5) Reverse turn (166–285 s, $\omega_{2}\approx -0.0087\;\mathrm{rad}/s$); (6) Final steady state (286–300 s). In order to evaluate the stability of the proposed algorithm in a complex underwater environment, this section injects two bursts of consecutive outliers into the positioning measurements. Each burst consists of four consecutive outlier points, with only one valid positioning point separating the two groups. This configuration is designed to examine the filter’s robustness in scenarios where reliable observations are nearly completely lost over an extended period.

#### 4.2.2. Result Analysis for Case 1

**Figure 3:**
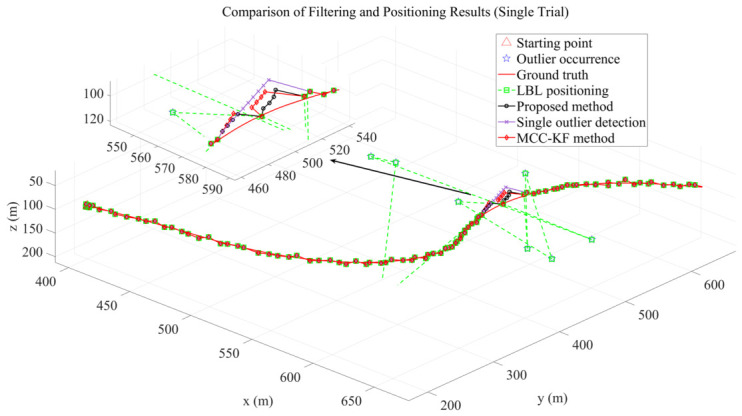
Comparison of positioning filtering trajectories and the ground truth for Case 1 (σ=1.5 m).

Figure 3 presents the filtering and positioning trajectories of the evaluated algorithms alongside the ground truth for a single trial of Case 1. During outlier occurrences, the LBL positioning results deviate from the ground truth, characterized by spatial jumps. Traditional single outlier detection methods yield visible trajectory deviations, primarily due to their inability to distinguish normal positioning points during consecutive abnormal observations. Although the MCC-KF method reduces these deviations compared to the single outlier detection approach, a noticeable offset from the ground truth remains when consecutive outliers are present, as detailed in the inset.

The proposed method aligns more closely with the ground truth throughout the trajectory. While consecutive anomalies tend to degrade the tracking performance of traditional filters and the MCC-KF, the proposed algorithm identifies the specific timestamps associated with both outliers and valid observations, which helps stabilize the state estimation.

**Figure 4:**
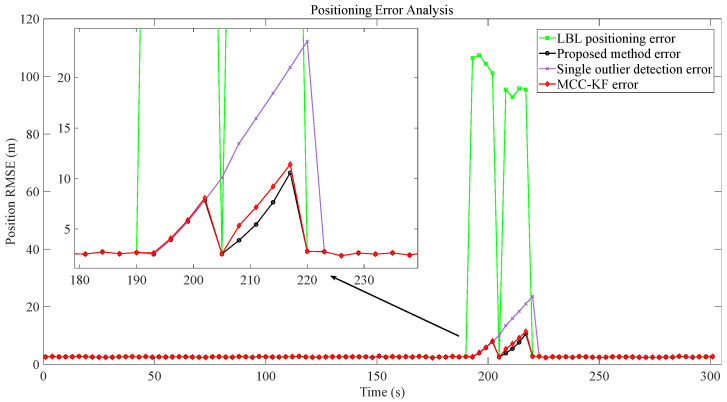
Temporal evolution of the total position RMSE for Case 1 (κ=100,σ=1.5 m).

Figure 4 presents the position root mean square error (RMSE) based on 100 Monte Carlo runs for Case 1. Between 190 s and 222 s, the raw LBL positioning error increases significantly due to two sequential outlier bursts. While the traditional single outlier detection method misclassifies the valid measurement point at approximately 205 s as an outlier—causing its RMSE to rise steadily to about 22 m—both the MCC-KF and the proposed method successfully recognize this point and return the positioning error to the baseline level of approximately 2.5 m. Throughout the second outlier interval (205–220 s), the RMSE of the proposed algorithm remains slightly lower than that of the MCC-KF, indicating that the proposed algorithm preserves a state estimation consistency comparable to that of the MCC-KF under prolonged contiguous outlier disturbances.

The convergence behavior after the outliers cease (t>220 s) further highlights the operational differences among the evaluated methods. Both the proposed method and the MCC-KF return to a steady state. In comparison, the traditional single outlier detection method exhibits a slight recovery lag, as it misidentifies the first normal measurement after the outlier block as anomalous. By isolating corrupted measurements while maximizing the utilization of valid observations, the proposed approach preserves higher positioning consistency during both anomaly periods and transition phases.

**Figure 5:**
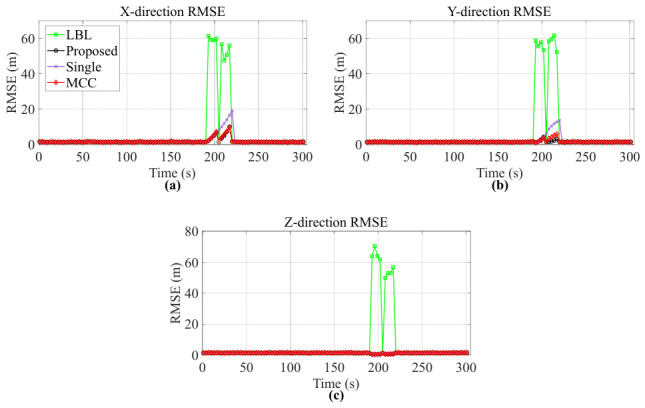
RMSE decomposition for Case 1 at σ=1.5 m: (**a**) X-direction, (**b**) Y-direction, and (**c**) Z-direction.

Figure 5 shows the decomposition of the position RMSE into its X, Y, and Z components for Case 1. The error profiles in the X and Y directions align with the overall trends shown in Figure 4, with the proposed algorithm maintaining the lowest RMSE among the evaluated filtering methods. In the Z direction, the RMSE values for the three filtering algorithms exhibit minimal fluctuation and remain close to the baseline. This steady performance is attributed to the relatively constrained motion of the underwater vehicle in the depth direction, which enables the state estimators to predict this state variable with high accuracy.

**Figure 6:**
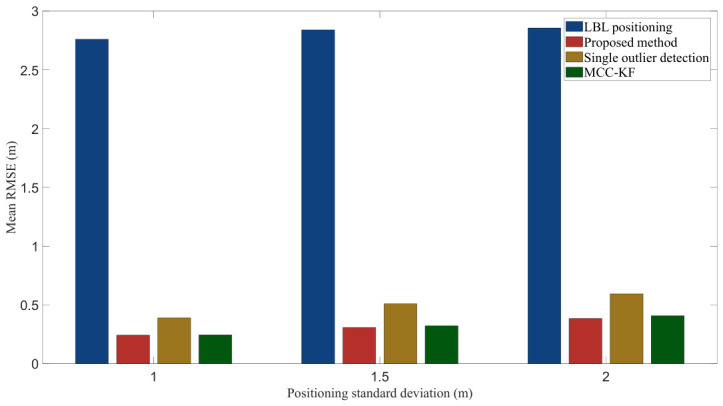
Comparison of ARMSE under different measurement noise standard deviations (σ∈{1,1.5,2} m) for case 1.

Figure 6 presents the mean RMSE for each algorithm under various measurement noise levels based on 100 Monte Carlo runs. The bar chart displays the positioning standard deviation (1 m, 1.5 m, and 2 m) along the horizontal axis and the mean RMSE along the vertical axis. Across all noise levels, raw LBL positioning yields the highest error, followed by the single outlier detection method. Although the MCC-KF method reduces the positioning error compared to these two approaches, the proposed method exhibits the lowest mean RMSE, remaining below 0.5 m under all evaluated noise levels.

### 4.3. Simulation and Result Analysis for Case 2

#### 4.3.1. Case 2 Scenario Description and Outlier Configuration

Case 2 is designed to evaluate the algorithm’s applicability under high maneuvering conditions. The turn rates and ranges are significantly increased compared to Case 1, with the total duration extended to 450 s. The phases include: (1) Stationary (0–15 s); (2) Acceleration (16–30 s); (3) CV (31–60 s); (4) $180^{{}^{\circ}}$ turn (61–180 s, $\omega_{1}\approx 0.026\;\mathrm{rad}/s$); (5) $-360^{{}^{\circ}}$ turn (181–390 s, $\omega_{2}\approx -0.029\;\mathrm{rad}/s$); (6) Final CV motion. In this Case, to evaluate the proposed algorithm’s adaptability to diverse situations, three sets of outliers with varying burst lengths—comprising a four-point sequence, a three-point sequence, and a single isolated point—are injected into the positioning measurement. This hybrid configuration is specifically designed to verify whether the KC-RIMM-KF logic can simultaneously suppress different types of outliers under high maneuvering conditions.

#### 4.3.2. Result Analysis for Case 2

**Figure 7:**
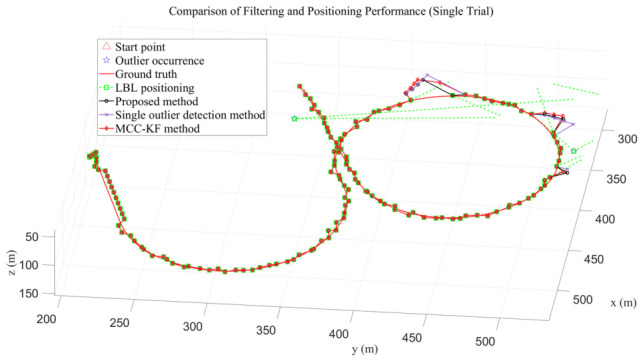
Comparison of positioning filtering trajectories for Case 2.

**Figure 8:**
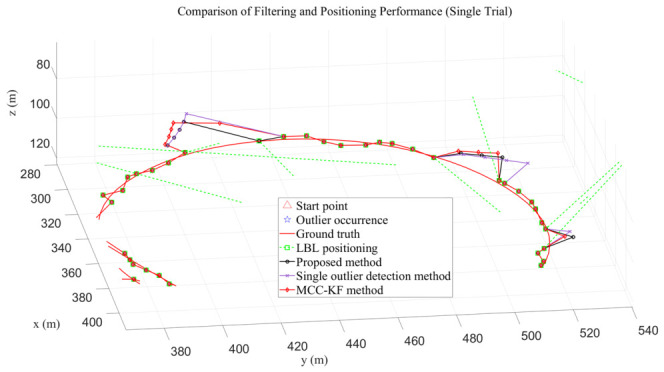
Zoomed-in view of the positioning filtering trajectories for Case 2.

Figure 7 and Figure 8 present the positioning trajectories and their corresponding local magnifications for Case 2. In the presence of spatially sequential outliers, the conventional single outlier detection approach exhibits noticeable deviations because its logic is primarily designed for isolated anomalies. The MCC-KF method maintains a relatively stable trajectory overall; however, when encountering a block of four consecutive outliers, it misclassifies an additional valid measurement, resulting in a localized tracking offset. The proposed method tracks the ground truth curve more closely, demonstrating consistent tracking accuracy by correctly identifying valid positioning points even immediately following sequential anomalies.

**Figure 9:**
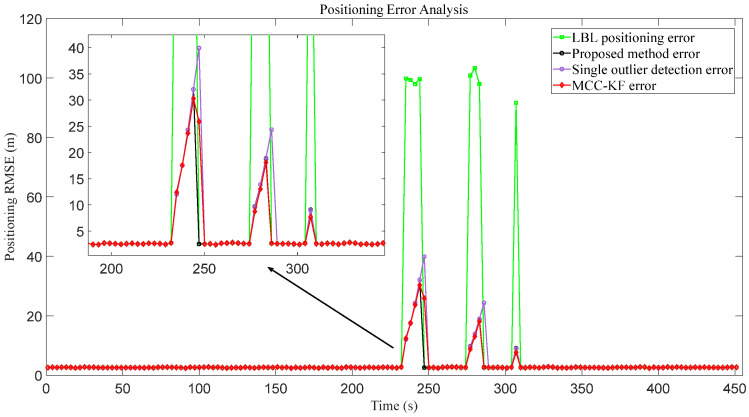
Temporal evolution of total position RMSE for Case 2.

Figure 9 shows the variation in the position RMSE over time for Case 2. Between 235 s and 310 s, the introduction of three distinct groups of outliers—comprising a 4-point sequence, a 3-point sequence, and a single-point outlier—causes the raw LBL positioning error to exceed 100 m. Comparative analysis indicates that while the evaluated filtering methods effectively suppress the isolated single-point outlier at approximately 307 s, their tracking performance varies when encountering consecutive outlier sequences.

As shown in the magnified inset, the traditional single outlier detection method exhibits detection lag for both consecutive outlier sequences. This approach fails to identify the termination point of sequential outliers, misclassifying the first normal positioning point immediately following the sequence as an anomaly, which introduces additional error and delays baseline recovery. Although the MCC-KF and the proposed method yield comparable RMSE values during most of the tracking process, a distinct difference is observed at the termination of the 4-point outlier sequence. Specifically, at 248 s, the MCC-KF method misidentifies the first normal positioning point as an outlier, resulting in a localized detection lag. This limitation is primarily associated with the use of a fixed kernel bandwidth in the conventional MCC-KF, which restricts its adaptability during rapid transitions between contiguous anomalies and valid measurements. Although adaptive kernel bandwidth MCC-KF variants reported in the literature can mitigate such detection lags and offer improved robustness, this study employs the standard fixed-bandwidth version as a representative baseline. The proposed algorithm correctly distinguishes this boundary, returning the estimation error to the baseline level immediately after the outlier sequence ceases.

**Figure 10:**
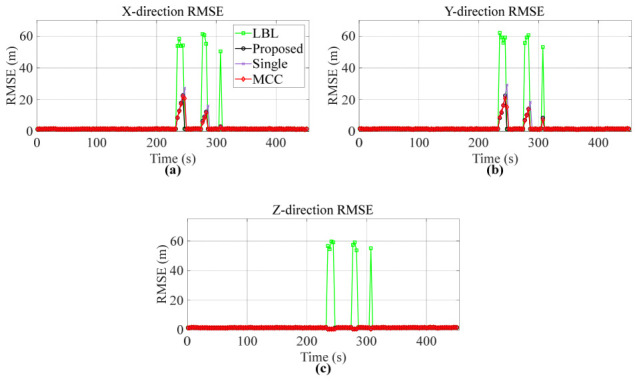
RMSE decomposition for Case 2 at σ=1.5 m: (**a**) X-direction, (**b**) Y-direction, and (**c**) Z-direction.

Figure 10 shows the decomposition of the position RMSE into its three coordinate components, illustrating the contribution of each axis to the overall positioning error. As shown in subplots (a) and (b), the error profiles in the X and Y directions are highly similar and constitute the primary sources of the total RMSE. During the sequential outlier periods starting at 235 s and 275 s, the single outlier detection method exhibits notable error increases, indicating error accumulation in the horizontal plane. Compared with the traditional approach, the MCC-KF method effectively suppresses the horizontal errors, but still exhibits localized detection lag. In subplot (c), the Z-direction errors for all three filtering algorithms remain low, representing an insignificant contribution to the overall RMSE.

**Figure 11:**
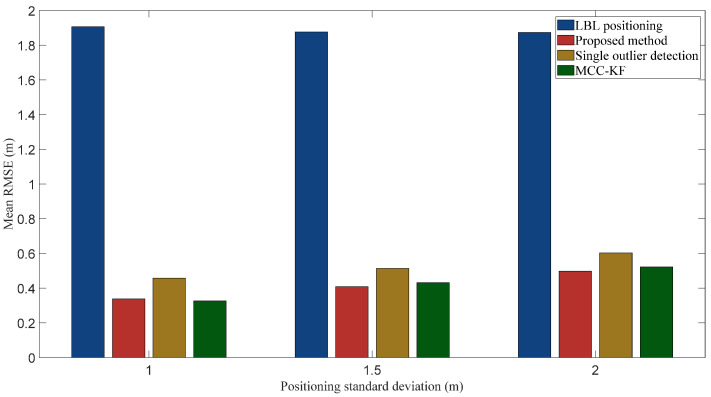
Comparison of ARMSE under different measurement noise standard deviations (σ∈{1,1.5,2} m) for case 2.

Figure 11 presents the mean RMSE results for Case 2 under varying measurement noise levels based on 100 Monte Carlo runs. Similarly to the trends observed in Case 1, both the proposed method and the MCC-KF algorithm maintain low positioning errors, with their mean RMSE values remaining within a narrow range of 0.3–0.52 m across all evaluated noise levels. By comparison, the raw LBL positioning and the single outlier detection method yield larger statistical errors. This consistency indicates the applicability of the proposed algorithm, showing that its outlier suppression capability remains effective across varying outlier patterns and sequence lengths.

### 4.4. Parameter Sensitivity and Robustness Analysis

The performance of the proposed outlier detection method is primarily determined by two key parameters: the maximum velocity threshold $v_{\max}$ and the anchor window size N. To justify the selection of these parameters and evaluate their impact on positioning accuracy, a sensitivity analysis was performed. This analysis also discusses the behavior of the algorithm when the outlier burst length exceeds the theoretical detection limits.

The parameter $v_{\max}$ represents the physical velocity limit of the underwater vehicle. Based on typical operational profiles and established literature [26], the nominal maximum velocity of the vehicle in this study is approximately 10 knots, which serves as the baseline parameter ($v_{\max}=10\;\mathrm{kn}$). To evaluate the sensitivity of the algorithm to this threshold, simulations were conducted by varying $v_{\max}$ to 4 kn and 12 kn while keeping other parameters constant.

**Figure 12:**
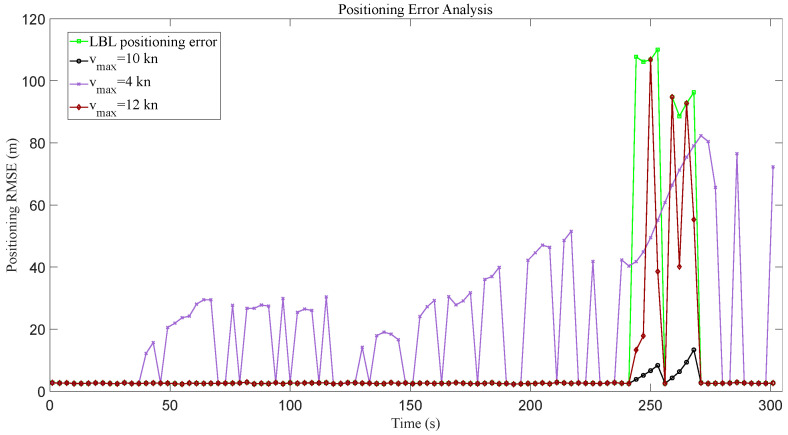
Positioning RMSE comparison under different velocity thresholds $v_{\max}$.

Figure 12 illustrates the resulting root mean square error (RMSE) profiles. When $v_{\max}$ is increased to 12 kn, the detection threshold becomes wider, reducing the sensitivity of the algorithm to anomalous deviations. This adjustment causes some outlier data points with smaller amplitudes to be misclassified as normal positions, leading to an increased RMSE in the interval where outliers occur. Conversely, when $v_{\max}$ is reduced to 4 kn, the constraint becomes overly restrictive relative to the actual dynamics of the vehicle. Under this configuration, normal positioning variations caused by standard measurement noise are incorrectly identified as outliers. This high false-alarm rate degrades the positioning accuracy throughout the entire trajectory, as evidenced by the consistently elevated RMSE even in outlier-free intervals. These results demonstrate that setting $v_{\max}$ near the actual physical limit of the vehicle provides a balanced trade-off between outlier suppression and false-alarm prevention.

Regarding the sensitivity of the anchor window size N, the analysis focuses on the algorithm’s performance when the outlier burst length exceeds the theoretical limit. Theoretically, the method is designed to identify a maximum of N−1 consecutive outliers successfully. To evaluate this boundary condition, a challenging scenario was designed containing two blocks of four consecutive outliers separated by a single normal positioning point.

**Figure 13:**
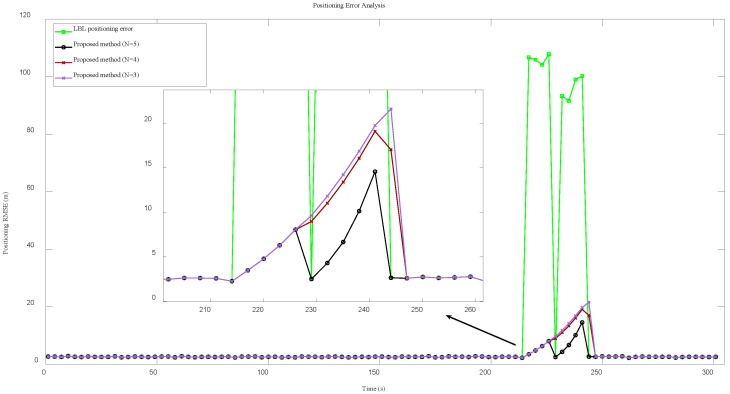
Positioning RMSE comparison under different numbers of anchor points N.

Figure 13 presents the positioning RMSE for different values of N (N=5,4,3). With N=5, the theoretical detection limit is N−1=4 consecutive outliers, which matches the maximum burst length in the simulation. The results indicate that the algorithm identifies all outliers in this scenario, maintaining a low RMSE. When N is reduced to 4, the maximum detectable consecutive outlier length decreases to 3. Because the outlier burst length of 4 exceeds this limit, the algorithm cannot fully resolve the anomalous sequence, causing an increase in the positioning error. If N is further reduced to 3, the theoretical detection capability is limited to 2 consecutive outliers, resulting in a more pronounced degradation in tracking performance and higher RMSE peaks. This comparison confirms that in practical applications, the window size N must be chosen such that the threshold N−1 is greater than or equal to the maximum expected length of consecutive outlier bursts in the deployment environment.

## 5. Conclusions

This paper presents a Kinematic-Constrained Robust Interacting Multiple Model Kalman Filter (KC-RIMM-KF) algorithm. By using initial stable positioning data to construct the target’s trajectory and integrating kinematic constraints with historical positioning data for outlier detection, the algorithm addresses the challenges posed by continuous, large-magnitude outliers in underwater LBL positioning systems. Based on Monte Carlo simulations across various maneuvering scenarios and noise intensities, the following conclusions are drawn:

First, the proposed algorithm achieves timely baseline recovery following outlier disappearance, effectively mitigating the false detections and detection lag typical of traditional schemes. Second, the algorithm demonstrates stable tracking performance under consecutive outlier conditions and diverse maneuvering scenarios. Third, both the KC-RIMM-KF and the MCC-KF maintain low positioning RMSE values across varying measurement noise levels, exhibiting improved error suppression compared to standard single-outlier detection methods. In summary, the KC-RIMM-KF enhances positioning accuracy and robustness under persistent anomalies, representing a reliable approach for underwater LBL positioning applications. While the present work establishes the fundamental framework under representative operational envelopes, deploying the system in highly unpredictable marine environments requires further adaptation. Consequently, our subsequent research will expand to investigate more complex, coupled phenomena—such as extreme beacon dropouts, spatiotemporal sound speed variations, and heavy-tailed non-Gaussian acoustic noise—to bridge the gap toward practical engineering applications.

## Figures and Tables

**Table 1:** Total filtering execution times and mission durations of the evaluated algorithms.

Test Group	Case 1 (s)	Case 2 (s)	MCC-KF Case 1 (s)
Run 1	0.022686	0.034464	0.005684
Run 2	0.024699	0.034048	0.006247
Run 3	0.022259	0.037374	0.005471
Entire mission time	300	450	300

## Data Availability

The raw data supporting the conclusions of this article will be made available by the authors on request.

## Abbreviations

The following abbreviations are used in this manuscript:

ARMSE	Average Root Mean Square Error
AUV	Autonomous Underwater Vehicle
CT	Coordinated Turn
CV	Constant Velocity
IMM	Interacting Multiple Model
IMM-KF	Interacting Multiple Model Kalman Filter
KC-RIMM-KF	Kinematic-Constrained Robust IMM-KF
LBL	Long Baseline
LS	Least Squares
MCC-KF	Maximum Correntropy Criterion Kalman Filter
OWTT	One-Way Travel Time
PDF	Probability Density Function
RMSE	Root Mean Square Error
UUV	Unmanned Underwater Vehicle
